# Immune Checkpoint Inhibitor-Induced Hypophysitis and Patterns of Loss of Pituitary Function

**DOI:** 10.3389/fonc.2022.836859

**Published:** 2022-03-08

**Authors:** Shlomit Jessel, Sarah A. Weiss, Matthew Austin, Amit Mahajan, Katrina Etts, Lin Zhang, Lilach Aizenbud, Ana Luisa Perdigoto, Michael Hurwitz, Mario Sznol, Kevan C. Herold, Harriet M. Kluger

**Affiliations:** ^1^ Department of Medicine (Medical Oncology), Yale University School of Medicine, New Haven, CT, United States; ^2^ Department of Radiology and Biomedical Imaging, Yale University School of Medicine, New Haven, CT, United States; ^3^ Department of Medicine (Endocrinology), Yale University School of Medicine, New Haven, CT, United States

**Keywords:** hypophysitis, immune checkpoint inhibitors (ICI), immune-related adverse events, melanoma, endocrinopathy

## Abstract

**Background:**

Immune checkpoint inhibitors (ICI) are clinically active across multiple tumor types but the associated immune-related adverse events (irAEs) lead to treatment delays or discontinuation and negatively impact quality-of-life. Hypophysitis is often a permanent irAE that may affect multiple pituitary hormonal axes. Here we comprehensively characterize our institution’s clinical experience with ICI-induced hypophysitis and the associated patterns of pituitary function loss.

**Methods:**

Patients with solid tumors, mostly melanoma and renal cell carcinoma (RCC), treated with ICI at Yale Cancer Center were prospectively enrolled from October 2016-May 2021. Demographics and clinical data were obtained from the medical record including type and timing of irAEs. Patients were included in this cohort if hypophysitis was diagnosed by pre-specified biochemical and clinical parameters.

**Results:**

The overall incidence of hypophysitis was 69/490 (14%) in patients with melanoma (n=58, 84%), RCC (n=10,14%), and merkel cell carcinoma (n=1, 1%) who received ipilimumab plus nivolumab (77%; 53/69), anti-PD-(L)1 (17%; 12/69), or ipilimumab monotherapy (6%; 4/69). Of the 69 patients analyzed, median time to hypophysitis on combination ICI versus anti-PD-1 was 2.8 vs. 4.1 months. The incidence of hypophysitis in patients with melanoma was 25% (46/187) with ipilimumab plus nivolumab and 5% (7/129) with anti-PD-(L)1 compared to 9% (7/77) and 8% (3/37), respectively, in patients with RCC. Patients who developed hypophysitis on combination ICI had a higher rate of headache (p=0.05) and co-occurring irAEs (p=0.01) compared anti-PD-(L1)1 monotherapy. At a median follow-up of 2.2 years, 77% of patients were alive. Objective response rates to ICI in melanoma patients were higher than previously reported for unselected populations. Central hypothyroidism and hypogonadism were the most common pituitary axes affected after the adrenal axis. In select cases, there was evidence of spontaneous rebound in free testosterone levels after an initial decline.

**Conclusions:**

We demonstrate a higher rate of ICI-induced hypophysitis than previously reported, which may be reflective of real-world practice due to increased awareness as experience with ICI has grown. In select cases, there was evidence of rebound in free testosterone and/or gonadotropins but not in adrenal axis hormones.

## Introduction

Monoclonal antibodies against PD-(L)1 and CTLA-4 have revolutionized cancer treatment and are FDA-approved for numerous oncologic indications for both unresectable disease and as adjuvant therapy for resected disease. Although immune checkpoint inhibitors (ICI) are clinically active in a proportion of patients across multiple tumor types, not all patients respond. A second major limitation of ICI is the unpredictable development of immune mediated adverse events (irAEs) which can negatively impact quality of life and often lead to treatment delays or discontinuation. The vast majority of irAEs can be managed with immunosuppressants, most commonly corticosteroids, however rare fatalities secondary to refractory irAEs have been reported (1). This is particularly challenging in the adjuvant setting, when many patients are already surgically cured. Prompt recognition and management of irAEs have improved as clinical experience with ICI has grown. However, organ-specific characterization of these irAEs including the pathogenesis, molecular and immunologic determinants, patient susceptibility, and association with anti-tumor immunity is less understood, and no predictive biomarkers are available.

Skin, gastrointestinal, and hepatic irAEs are generally the most common, but virtually any organ can be impacted by ICI either during the course of treatment or occasionally months to years after treatment has been stopped (2, 3). The majority of irAEs are reversible once promptly diagnosed and managed however a small percentage, most commonly endocrinopathies, are long-lasting or irreversible and can require long-standing replacement therapy. Several studies have suggested a link between immune-mediated endocrinopathies and survival including thyroid disease (4–6), but these findings are not necessarily generalizable and may depend on the ICI regimen, tumor type, and the affected organ (7). After thyroid dysfunction, hypophysitis is the second most common immune-mediated endocrinopathy. The diagnosis is made based upon a combination of pituitary and effector hormone laboratory abnormalities, clinical symptoms, and radiographic findings of pituitary inflammation. In clinical trials of patients with advanced melanoma, the incidence of immune-mediated hypophysitis has differed based on the ICI regimen. The incidence of hypophysitis has ranged from 1-18% in patients treated with ipilimumab (8–13), 0.5-1.5% for PD-1 inhibitors (2, 12, 14), and up to 13% for combination therapy with ipilimumab plus nivolumab (10, 15, 16). Of note, not all of these studies mandated measurement of pituitary hormones, and the real-world incidence of hypophysitis might in fact be higher. For example, a case series of patients with melanoma treated with ICI report hypophysitis in an estimated 10% on combination therapy and 5% on anti-PD-1 (17). However, most case series report on anti-PD-1 alone rather than ipilimumab plus nivolumab and others lack a denominator to be able to reliably calculate the true incidence of hypophysitis (18, 19). Inflammation that affects adrenocorticotropic hormone (ACTH) producing cells causing central adrenal insufficiency is most common, but disruptions to other hormonal pathways can occur, for example resulting in central hypothyroidism and hypogonadotropic hypogonadism (17). The purpose of this study is to comprehensively characterize the clinical experience of ICI-induced hypophysitis and patterns of pituitary function loss from a single institution.

## Methods

Patients treated with ICI in Yale Cancer Center’s Melanoma and Renal Cell Carcinoma (RCC) Programs were prospectively enrolled on protocol #0608001773, approved by the Yale University Institutional Review Board. The objectives of this protocol are to characterize irAEs and identify biomarkers and mechanisms of immunologic responses that contribute to irAEs in cancer patients treated with ICI. Key criteria for inclusion were adults with melanoma, renal cell carcinoma, and merkel cell carcinoma treated with ICI which are the tumor types seen in our practice. Written informed consent was obtained from all subjects. Demographic, clinical, radiographic, and pathologic data including tumor type, ICI regimen, response to treatment, and survival status were recorded for each subject and were available by review of the electronic medical record. The date, duration, and type of each irAE that developed during the course of ICI treatment, including hypophysitis, was documented for each patient. Serial blood draws for research were performed, when possible, pre-treatment at cycles 1 through 4 and every 3 months thereafter and/or at the time of ICI-induced toxicity and while on steroids. Blood samples were processed and stored in our dedicated biorepository.

Patients were included in this analysis if they had evidence of hypophysitis defined by: 1) suggestive clinical symptoms such as fatigue, headache, nausea or vomiting; and 2) biochemical parameters consistent with pituitary hormone deficiency which included low cortisol and abnormalities in the following hormones, as clinically indicated: ACTH, thyroid stimulating hormone (TSH), free T4, luteinizing hormone (LH), follicle stimulating hormone (FSH), and testosterone in males.

Central hypothyroidism was defined as low or low-normal free T4 and low or inappropriately normal TSH. Central hypogonadism in men was defined as low testosterone and low or inappropriately normal LH. Free testosterone levels were measured by ELISA (ALPCO, #11-FTSHU-E01, Salem, NH, USA) on available serum from male patients who developed hypophysitis including at baseline, time of hypophysitis diagnosis, and post-hypophysitis, when available. Expected normal free testosterone values from this assay are 5.7-30.7 pg/mL for males between the ages of 40 to 59 years old and 5.9-27 pg/mL for males ≥ 60 years old. The lower limit of detection for the test is 0.018 pg/mL. LH was measured on the same samples by ELISA (ALPCO, #11-LUTHU-E01, Salem, NH, USA). The normal LH range for males is 1.5-9.3 IU/L. The lower limit of detection for this assay is 0.2 IU/L.

Brain MRIs that were performed within 1 month of development of hypophysitis were retrospectively re-reviewed by an independent radiologist (A.M.) for pituitary enlargement. Statistical methods were descriptive. All patients were analyzed from start of treatment until the data cut-off of July 19, 2021. For comparison of clinical features between the patients treated with ipilimumab plus nivolumab versus anti-PD-1 alone, t-tests were used to compare means between the groups and the Chi Square test was used for categorical variables. Best overall response (BOR) was defined as the best response [complete response (CR), partial response (PR), stable disease (SD), or progressive disease (PD)] documented on at least two consecutive imaging studies from the start of the ICI regimen on which the patient developed hypophysitis.

## Results

### Patient Characteristics and Incidence of Hypophysitis

Between October 2016 and May 2021, 490 patients receiving ICI were enrolled on the protocol with the majority having melanoma (n=320), renal cell carcinoma (n=115), or merkel cell carcinoma (n=12). Several other cancer types were also included: biliary tract carcinoma (n=2), basal cell carcinoma (n=3), breast (n=7), lung (n=14), colon (n=3), gastroesophageal (n=4), pancreatic (n=2), prostate (n=3), rectal (n=1), and cutaneous squamous cell carcinoma (n=4). Sixty-nine out of 490 patients on ICI (14%) developed hypophysitis. Our analysis focuses on these 69 patients with hypophysitis. The tumor types represented in this cohort were reflective of the patients who were treated in the Yale Melanoma and RCC Programs and included melanoma (n=58, 84%), RCC (n=10,14%), and merkel cell carcinoma (n=1, 1%). By ICI regimen, hypophysitis developed in 19% (53/277) of patients who received ipilimumab plus nivolumab and in 6% (12/212) of patients who received anti-PD-1. The sample size of patients who received ipilimumab only was too small to calculate the hypophysitis incidence. For patients who received ipilimumab plus nivolumab, there was a higher incidence of hypophysitis in those with melanoma (25%; 46/187) versus RCC (9%; 7/77) (p=0.004). Of note, patients with melanoma generally received ipilimumab at 3 mg/kg instead of 1 mg/kg, the standard of care for RCC. Incidence of hypophysitis on anti-PD-1 monotherapy was 5% (7/129) in patients with melanoma and 8% (3/37) in patients with RCC. Ten percent (1/10) of patients with Merkel cell carcinoma treated with anti-PD-1 developed hypophysitis. Demographics and clinical characteristics of these patients are outlined in **Table 1**. The majority of patients were male (68%). Median age at the time of development of immune-mediated hypophysitis was 64 years.

**Table 1:** Baseline clinical characteristics.

Characteristic	N=69	%
**Gender**		
Male	47	68%
Female	22	32%
**Age at time of hypopituitarism**		
Median	64	–
Range	32-83	–
**Tumor type**		
Melanoma	58	84%
Renal cell carcinoma	10	14%
Merkel cell carcinoma	1	1%
**Systemic Therapy Regimen at time of Hypophysitis**		
Ipilimumab and Nivolumab	53	77%
Anti-PD-(L)1	8	12%
Ipilimumab	4	6%
Anti-PD-(L)1 + Investigational Drug	4	6%
**Prior Systemic Therapy**		
Yes	21	30%
No	48	70%
**Prior ICI**		
Yes	17	25%
No	52	75%

### Timing and Type of Therapy

The majority of patients were treatment-naive (71%), and 17 out of 20 with prior therapies had received prior ICI. At the time hypophysitis developed, patients were receiving ipilimumab plus nivolumab (77%; 53/69), anti-PD-(L)1 monotherapy (12%; 8/69), anti-PD-(L)1 with the addition of an investigational agent (6%; 4/69), or ipilimumab monotherapy (6%; 4/69) (**Table 1**). Median time to hypophysitis diagnosis from the start of the ICI regimen was 95 days (range 23-523) and after 4 cycles of therapy (range 1-18). Median time to hypophysitis differed by ICI regimen, occurring earlier for patients who received ipilimumab plus nivolumab (2.8 months) compared to anti-PD-1 monotherapy (4.1 months) (p=0.0006) (**Table 1**). There were several cases of delayed development of hypophysitis (**Supplementary Figure 1**). Eight patients (12%) developed hypophysitis 6-12 months after starting their ICI regimen with 6 out of 8 having received ipilimumab plus nivolumab. Three patients (4%) developed hypophysitis after 1 year. Two had received anti-PD-(L)1 and 1 received ipilimumab. The longest time to development of hypophysitis was 523 days in a patient with RCC treated with atezolizumab plus bevacizumab.

### Clinical Presentation

All patients were symptomatic at the time hypophysitis was diagnosed. The most common presentations were fatigue (86%), headache (43%), nausea and/or vomiting (39%), hypotension (7%), and/or visual changes (4%) (**Table 2**). Patients who received ipilimumab plus nivolumab were more likely to experience a headache at time of hypophysitis diagnosis compared to those treated with anti-PD-(L)1 monotherapy (47% vs. 17%, p=0.05), but there were no significant differences in frequency of nausea and/or vomiting, hypotension, or fatigue between the groups.

**Table 2:** Time to hypophysitis diagnosis, presenting symptoms, and co-occurring irAEs.

Characteristic	Ipi+Nivon=53 (%)	Anti-PD-(L)1n=12 (%)	Ipin=4 (%)	p-value*
**Timing**				
Median ICI cycles until hypophysitis (range)	4 (1-9)	6.5 (2-13)	4.5 (3-7)	**<0.0001**
Median days on ICI until hypophysitis (range)	84 (23-259)	124 (44-523)	132 (91-399)	**0.0006**
**Presenting symptom(s)**				
Fatigue	46 (87)	10 (83)	3 (75)	0.75
Headache	25 (47)	2 (17)	3 (75)	**0.05**
Nausea/vomiting	19 (36)	6 (50)	2 (50)	0.36
Hypotension	3 (6)	2 (17)	0 (0)	0.20
**Other irAE(s)^¥^**				
Any	44 (83)	6 (50)	2 (50)	**0.01**
**Co-occurring thyroid dysfunction**				
Primary Hypothyroidism	9 (17)	1 (8)	1 (25)	0.76
Transient hyperthyroidism without subsequent hypothyroidism	6 (11)	0 (0)	0 (0)	NA
Central Hypothyroidism	19 (36)	2 (17)	3 (75)	0.2

*p-values comparing ipi+nivo to anti-PD-1.

^¥^Excludes cases of hypothyroidism secondary to hypophysitis; at any point on ICI therapy.

Bold denotes statistically significant.

Co-occurring irAEs, defined as irAEs other than hypophysitis that developed within a 3-month window from the time of hypophysitis diagnosis, developed in 46% (32/69) of patients (**Table 3**) but development of irAEs at any timepoint during ICI therapy were more common in patients who received ipilimumab plus nivolumab compared to anti-PD-1 alone (83% vs. 50%, p=0.01) (**Table 2**). The most common additional irAEs were colitis (35%), rash (25%), and hepatitis (25%). Other endocrinopathies that developed were thyroid dysfunction, which is discussed separately below, and ICI-induced diabetes in three patients (4%). Three additional patients who had pre-existing type II diabetes at baseline experienced worsening of their diabetes while on ICI and became insulin-dependent. All six of these patients were treated with ipilimumab plus nivolumab. At any point in the treatment course, 75% of patients developed at least 1 additional irAE, excluding central hypothyroidism secondary to hypophysitis (**Table 3**). Half of patients who received anti-PD-(L)1 therapy had hypophysitis as their only irAE compared to only 17% of patients on ipilimumab plus nivolumab (p=0.01).

**Table 3:** Clinical presentation of hypophysitis and association with other irAEs.

Clinical Presentation		
Fatigue	59	86%
Headache	30	43%
Nausea/Vomiting	27	39%
Hypotension	5	7%
Visual Changes	3	4%
**Co-occurring irAEs within 3 months**		
Yes	32	46%
No	37	54%
Other irAEs by organ system*		
Colitis	24	35%
Rash (poorly documented)	17	25%
Hepatitis	17	25%
Primary Hypothyroidism	11	16%
Arthritis	6	9%
Isolated thyroiditis	6	9%
Pneumonitis	6	9%
Pancreatitis	4	6%
Nephritis	4	6%
Sicca	4	6%
Ocular Toxicity	4	6%
Diabetes	3	4%
Neurotoxicity	2	3%
Myositis	2	3%
**Number of co-occurring organ-specific irAEs**		
0 (hypophysitis only)	17	25%
1	21	30%
2	16	23%
3	8	12%
4	5	7%
5	1	1%
8	1	1%

*Cases of hypothyroidism secondary to hypophysitis are not included.

### Clinical Outcomes

Median follow-up time, defined as from the date of hypophysitis diagnosis to death or last follow-up, was 2.2 years. Sixty-seven patients received treatment for advanced disease while only two were on adjuvant anti-PD-1. For the 46 patients with unresectable melanoma who received ipilimumab plus nivolumab, BOR was CR (n=18; 39%), PR (n=15; 33%), SD (n=3; 7%), and PD (n=10; 22%). In the 7 melanoma patients who received anti-PD-1, BOR was CR (n=4; 57%), PR (n=1; 14%), and PD (n=2; 29%). In the 10 patients with RCC, BOR was PR (n=4; 40%), SD (n=4; 40%), and PD (n=2; 20%). The one patient with Merkel cell carcinoma had progressive disease. At the data cut-off, 77% of patients were alive and 23% were deceased.

### Biochemical Analyses

**Figure 1:**
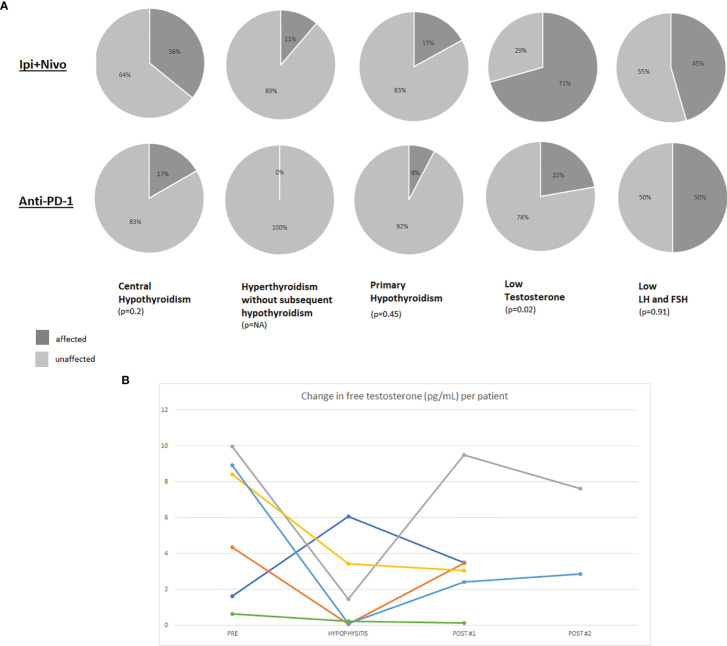
**(A)** Pie charts representing the percentage of hypophysitis patients who were affected by hormonal axis deficiencies. The denominator for each pie chart represents the number of patients who were evaluated with diagnostic laboratory tests for the particular axis since not certain hormones and their effectors were not routinely tested in all patients. Results are presented for patients who developed hypophysitis on ipilimumab plus nivolumab or anti-PD-(L)1. **(B)** Line graph demonstrating changes in free testosterone (pg/mL) per patient relative to the time of hypophysitis diagnosis. A standardized assay for free testosterone was performed on male patients with stored blood samples collected on our protocol.

**Figure 1A** demonstrates the proportion of patients with central hypothyroidism or hypogonadism, stratified by ICI regimen. Ipilimumab was not included in the Figure due to the low number of patients (n=4) on ipilimumab monotherapy in our cohort. By definition, serum cortisol was low in all 69 patients. ACTH was measured in 51% (35/69) of patients and was universally low. The low cortisol was associated with hyponatremia in 25% (17/69) of cases. There was only 1 case of hyperkalemia and while the initial ACTH was high in this case likely due to pituitary inflammation, the ACTH value subsequently became low and along with a low cortisol measurement, was reflective of a central process. The remainder of the hormones and/or their effectors were measured as clinically indicated.

#### TSH/Free T4

At the time of hypophysitis diagnosis, TSH was measured in 100% (69/69) of patients and free T4 was measured in 80% (55/69). Twenty-three (33%) patients were euthyroid and 5 patients (7%) had pre-existing hypothyroidism and were already on replacement therapy prior to the diagnosis of hypophysitis. Thyroid dysfunction was identified in 41/69 patients as: central hypothyroidism (35%; 24/69), primary hypothyroidism (16%; 11/69), and isolated hyperthyroidism without subsequent hypothyroidism (9%; 6/69). Central hypothyroidism, primary hypothyroidism, and isolated thyroiditis all occurred at higher rates in patients on ipilimumab plus nivolumab compared to anti-PD-1 monotherapy.

#### Testosterone

Total and/or free serum testosterone was measured in 20 of the 47 male patients (43%) as clinically indicated and was below the normal range in the majority (15/20; 70%). However these levels were not all obtained at the time of hypophysitis diagnosis. As part of our protocol, patients had blood available for analysis from several time points including pre-toxicity, time of hypophysitis diagnosis, and post-hypophysitis. Free testosterone, FSH and LH were tested at these timepoints from available serum (**Supplementary Table 1**). Out of 47 male patients with hypophysitis, twenty had research bloods available for analysis: 7 from pre- and post-hypophysitis diagnosis, 6 from pre, time of hypophysitis, and post; 6 at time of hypophysitis and post-hypophysitis. Two patients were excluded from the analysis for lack of interpretable timepoints (one patient had blood from before and at diagnosis of hypophysitis and the other only had samples from after diagnosis). Overall, samples were available to calculate change in free testosterone levels from prior to and after development of hypophysitis (n=7), prior to and at diagnosis of hypophysitis (n=5), and at and after the diagnosis of hypophysitis (n=10). Free testosterone decreased in 5 out of 7 patients from baseline to the time of hypophysitis diagnosis. In the 6 patients for which free testosterone measurements from all three timepoints were available, there were varying degrees of recovery of testosterone production after diagnosis of hypophysitis (**Figure 1B** and **Supplementary Figure 2**). Three of the six patients had a rebound in their free testosterone after developing hypophysitis. The rebound levels were obtained at 201, 371, and 394 days (mean: 268 days) post-hypophysitis. Since the timing of the samples was variable and distant from the diagnosis of hypophysitis, we are unable to report whether the free testosterone recovery occurred even sooner. LH decreased at time of hypophysitis in 2 of the 6 cases. All 6 patients had received ipilimumab plus nivolumab.

### Brain MRI Findings

Forty-nine patients (71%) had an MRI of the brain performed within 1 month (range -28 to +30 days; median -1 day) of developing hypophysitis, either due to neurologic symptoms or as a routine follow-up scan while on ICI (**Table 4**). Of the 49 patients with available brain imaging, 23 (47%) had radiographic evidence of pituitary enlargement that further supported the diagnosis of hypophysitis. Of the 23 patients with pituitary enlargement, 16 (70%) presented with headache. The majority of these patients (n=20; 87%) received ipilimumab plus nivolumab. Conversely, while not all patients with pituitary enlargement on imaging presented with headache, not all patients with a headache had evidence of pituitary enlargement. Of the 30 patients who presented with headache, 26 had MRI brain imaging available for review and 16 (62%) had radiographic evidence of pituitary enlargement. In patients treated with ipilimumab plus nivolumab, 20/41 (49%) available brain MRIs demonstrated evidence of pituitary enlargement versus only 1/5 (20%) in patients treated with anti-PD-(L)1 monotherapy (p=0.22).

**Table 4:** MRI brain imaging available within 1 month of clinical hypophysitis diagnosis.

	ALLn=49 (%)	Ipi+nivon=41 (%)	Anti-PD-(L)1n=5 (%)	Ipin=3 (%)
**Pituitary enlargement**	23 (47)	20 (49)	1 (20)	2 (67)
* With headache*	16 (70)	14 (70)	0 (0)	2 (100)
* Without headache*	7 (30)	6 (30)	1 (100)	0 (0)
**Timing of pituitary enlargement on imaging in relation to clinical hypophysitis diagnosis**				
* Pre-diagnosis*	11 (48)	9 (45)	1 (100)	1 (50)
* Day-of diagnosis*	8 (35)	8 (40)	0 (0)	0 (0)
* Post-diagnosis*	4 (17)	3 (15)	0 (0)	1 (50)
**Normal sized pituitary**	26 (53)	21 (51)	4 (80)	1 (33)
* With headache*	9 (35)	8 (38)	0 (0)	1 (100)

## Discussion

Here we report on our single institution experience with hypophysitis in patients treated with ICI, the majority of whom had melanoma. The incidence of hypophysitis in our cohort overall was 19% in patients treated with ipilimumab plus nivolumab and 6% for anti-PD-1, and highest for patients with melanoma treated with ipilimumab plus nivolumab (25%). Although these rates are higher than the incidence reported in ICI clinical trials for patients with melanoma (8, 10–12, 15, 20), they may be more reflective of real-world practice and an increased awareness and recognition of this irAE. Although the incidence of hypophysitis in melanoma patients treated with ipilimumab plus nivolumab may be even higher than expected, our series is one of the largest to report this.

Patients who developed hypophysitis from ipilimumab plus nivolumab compared to anti-PD-1 monotherapy tended to present earlier, usually within the first 3-4 months of ICI initiation, with higher rates of headache and co-occurring irAEs. Although most cases of hypophysitis arose early, three patients developed hypophysitis over 1 year after starting ICI. Case reports of late-onset hypophysitis presenting months after discontinuation of ICI have previously been reported (21, 22). Our data reinforce the importance of ongoing monitoring for irAE development beyond the ICI treatment period.

Regarding the presenting symptoms of hypophysitis, headache occurred more commonly in the combination ICI group compared to anti-PD-1 monotherapy (47% vs. 17%), often with pituitary enlargement on MRI, but not always, and rarely in patients on anti-PD-1 monotherapy, in support of prior studies (18). While MRI brain may be useful as a contributory data point for the evaluation of ICI-induced hypophysitis, the absence of pituitary enlargement is not diagnostic, and headache may still be an attributable symptom of hypophysitis in the absence of pituitary enlargement on MRI. Most of the MRIs performed on patients in this series were done for surveillance for brain metastasis, and the variable timing of brain imaging in relation to the clinical hypophysitis diagnosis in this cohort revealed that pituitary enlargement on MRI may occur before a biochemical diagnosis of hypophysitis is made. Incidental finding of pituitary enlargement on MRI brain in a patient receiving or previously on ICI should prompt a clinical and laboratory assessment for active or impending hypophysitis.

Almost half of patients experienced at least one co-occurring irAE and 75% developed another irAE at some point during their ICI treatment course, reinforcing that the diagnosis of hypophysitis should prompt an assessment for co-occurring irAEs and an awareness for future irAEs that may develop. For example, six patients developed insulin-dependent diabetes, whether presenting as new, acute-onset (n=3) or with worsening hyperglycemia in a background of pre-existing diabetes (n=3). ICI-induced diabetes is a rare but often permanent endocrinopathy requiring life-long insulin therapy. Other cases of ICI-induced diabetes and hypophysitis have been reported (23), including in 5% of patients with ICI-induced hypophysitis in the World Health Organization’s pharmacovigilance database (24) and reflective of our data here.

There are conflicting data on whether the development of irAEs is associated with improved clinical outcomes. In our cohort, after a median follow-up of 2.2 years, objective response rate (CR + PR) was 74% ((18 + 16)/46) for the melanoma patients who developed hypophysitis on ipilimumab plus nivolumab and 71% ((4 + 1)/7) for anti-PD-1, both of which are higher than that reported in the literature (25). At the time of data cut-off, the majority of patients remain alive. Although our study does not include a matched comparator arm of patients who did not develop hypophysitis, melanoma patients treated with ipilimumab plus nivolumab who developed hypophysitis as a whole had improved clinical outcomes. This conclusion is supported by a prior study of patients with ipilimumab-induced hypophysitis who had improved overall survival compared to patients without hypophysitis (26). Furthermore, a small study has also suggested that the development of pituitary-related irAEs in patients with melanoma treated with ICI is associated with prolonged overall survival compared to those who did not develop hypophysitis (27). The RCC cohort sample size is too small to draw similar conclusions from, but BOR of PR occurred in 40% (n=4) patients, 3 of whom received ipilimumab plus nivolumab and one who received atezolizumab plus bevacizumab.

While our protocol allows for enrollment of all patients treated with ICI whether in the neoadjuvant, adjuvant, or metastatic setting, only 2 of the 69 patients in this cohort who developed hypophysitis were on adjuvant therapy. Although this raises the question of whether the incidence of hypophysitis is less in the adjuvant setting compared to the metastatic setting, the denominator of patients on adjuvant therapy in our database was not available at this time. Of note, all adjuvant patients were treated with anti-PD-1 monotherapy. In CheckMate 238, the incidence of hypophysitis was 1.5% with adjuvant nivolumab and 10.6% with adjuvant ipilimumab in patients with resected stage IIB-IV melanoma (12). At the 4-year follow-up, late emergent TRAEs (voluntarily reported > 100 days after the last dose of ICI) were uncommon and hypophysitis was identified in <1% of patients in either adjuvant arm (28). Despite the low rate of hypophysitis in the adjuvant setting and with PD-1 inhibitors overall, ICI-induced endocrinopathies that develop are typically permanent and require life-long replacement therapy. This can affect quality-of-life and should be a consideration in the risk-benefit analysis when offering patients adjuvant ICI, particularly in patients with earlier stage IIB-IIIA disease or in those patients who have a BRAF V600 mutation and may be eligible for adjuvant targeted therapy instead, which does not pose a risk for permanent endocrinopathies.

Our study was not designed to determine whether the incidence of ICI-induced hypophysitis differs by tumor type since the majority of patients enrolled on our protocol had melanoma. We cannot draw broad conclusions regarding the small numbers of patients with hypophysitis who had RCC or merkel cell carcinoma. In a large meta-analysis studying ICI-induced endocrinopathies in patients with solid tumors, 85 out of 6472 patients developed hypophysitis, and among these 76 had melanoma (15). Interestingly, hypophysitis has not been reported in phase III trials of ipilimumab plus nivolumab for treatment-naïve clear cell RCC or for stage IV or recurrent non-small cell lung cancer. However, this may have been mostly impacted by the fact that only treatment-related AEs that occurred in >15% of patients were reported (29, 30) and this would likely exclude hypophysitis. The higher incidence in melanoma patients on ipilimumab plus nivolumab compared to RCC patients might reflect the difference in standard practice dosing, as the approved regimen for melanoma involves a higher dose of ipilimumab. It is also possible that there are specific antigens and epitopes uniquely expressed only in melanoma that are shared with otherwise normal pituitary tissue, resulting in cross reactivity from peripheral circulating T-cells.

Finally, there are limited data describing the impact of ICI on other hormonal axes during the development of hypophysitis. We demonstrate that central hypothyroidism occurs in roughly one-third of patients who develop hypophysitis on combination ICI and in almost 20% on anti-PD-1 monotherapy. Furthermore, low testosterone was identified in the majority of male patients with hypophysitis in whom it was checked. While age may be a contributing factor, hypogonadism may be an underrecognized and under-tested occurrence in association with hypophysitis. Testing is therefore warranted in men who develop hypophysitis especially if they report ongoing non-specific symptoms such as fatigue, depressed mood and/or libido, or more overtly, hot flashes. Interestingly, a small proportion of patients had an initial drop in free testosterone at the time of hypophysitis diagnosis with spontaneous rebound or recovery of free testosterone in the months after developing hypophysitis. There was no recovery of the adrenal axis, consistent with prior reports (31), as all patients who developed hypophysitis in this cohort remain on some level of steroid replacement although some doses were able to be lowered with time. Regarding the thyroid hormones, once central hypothyroidism developed, patients were kept on levothyroxine and attempts were not made to wean patients off this medication. Conversely, the gonadal axis appears to be capable of a certain degree of recovery in this cohort. There are limited data on recovery patterns, however in one published cohort, 63% (12/19) of patients who developed central hypogonadism in association with hypophysitis had spontaneous recovery at a median of 17 weeks (32). Another study of ipilimumab-induced hypophysitis reported resolution of central hypothyroidism and hypogonadism in 64% and 45% of studied cases, respectively (31).

While the mechanisms of ICI-induced endocrinopathies including hypophysitis and the precipitating factors are not fully elucidated, CTLA-4 polymorphisms have been associated with autoimmune endocrinopathies (33). The incidence of hypophysitis and radiographic evidence of pituitary enlargement is higher with ipilimumab-containing regimens, supported by the finding that CTLA-4 is expressed in the pituitary (34), and by murine studies demonstrating the development of anti-pituitary antibodies in mice treated with anti-CTLA-4 (35). Patients treated with ipilimumab who developed hypophysitis also had evidence of anti-pituitary antibodies targeting various cell types including thyrotrophs, corticotrophs, and gonadotrophs, but this differed by individual patient. It is still unclear if PD-1 or PD-L1 is expressed in the pituitary (33, 35).

We acknowledge several limitations to this analysis, including the single institution nature of the study and the non-uniformity of diagnoses and categories of ICI therapy. While cortisol is frequently checked in our clinical practice upon suspicion of hypophysitis, we did not prospectively measure cortisol levels on all patients, including those who are asymptomatic, so we do not know the true incidence of hypophysitis. Additionally, measurement of other pituitary axis hormones was variable. When checked, the blood was drawn at variable time points in relation to the timing of hypophysitis and the decision to check was often impacted by other clinical symptomatology and whether the patient was evaluated by an endocrinologist or not.

In conclusion, irAEs can cause significant impact to a patient’s quality of life and may necessitate treatment delays or discontinuation. Hypophysitis is typically an irreversible irAE that can be managed with maintenance steroid replacement, however in rare cases it can be life-threatening. Moreover, it can impact quality of life and fertility, and can pose a challenge with use of further therapies such as cytokine therapies. Studies to better understand how to predict development of irAEs, including hypophysitis, and which patients are most susceptible, are underway.

## Data Availability Statement

Availability of the dataset can be discussed on a case-by-case basis with the corresponding author. Requests to access the datasets should be directed to saweiss@cinj.rutgers.edu.

## Ethics Statement

The studies involving human participants were reviewed and approved by Yale University Institutional Review Board. The patients/participants provided their written informed consent to participate in this study.

## Author Contributions

SJ, SW, and HK contributed to conception and design of the study. SJ, MA, SW, KE, LZ, and LA contributed to and organized the database. Patients were recruited and cared for in the clinical practices of SW, HK, MS, MH, and KH where the clinical data was collected. SJ performed the testosterone and gonadotropin assays. AM reviewed available MRIs of the brain and assisted with interpretation of imaging data. KH and AP provided feedback on the specifics of presenting the endocrinopathies. SW wrote the first draft of the manuscript. All authors contributed to manuscript revision, read, and approved the submitted version.

## Funding

This study received funding from P30DK116577 (KH), R01 CA227473 (KH, HK), K12CA215110 (SW, AP, HK), and the Yale SPORE in Skin Cancer P50 CA121974 (HK). The funders were not involved in the study design, collection, analysis, interpretation of data, the writing of this article or the decision to submit it for publication.

## Conflict of Interest

Author MH has consulted for Bristol Myers Squibb, CRISPR Therapeutics, Exelixis, and Nektar Therapeutics; received research funds from Achilles Therapeutics, Apexigen, Arrowhead, Arvinas, Astellas, AstraZeneca, Bayer, Bristol Myer Squibb, CRISPR Therapeutics, Corvus, Eli Lilly, Endocyte, Genentech, Genmab, GSK, Innocrin, Iovance, KSQ, Merck, Nektar Therapeutics, Novartis, Pfizer, Progenics, Sanofi Aventis, Seattle Genetics, Tmunity, Torque, and Unum; and has another relationship with Gamida Cell and Arvinas. Author HK has consulted for: Nektar, Iovance, Immunocore, Celldex, Array Biopharma, Merck, Elevate Bio, Instil Bio, Bristol-Myers Squibb, Clinigen, Shionogi, Chemocentryx, Calithera, and Signatero. Author MS has consulted for Adaptimmune, Pfizer, Kadmon, Pierre-Fabre, Biond, Nextcure, Incyte, Alligator, Bristol-Myers, Ocellaris, Simcha, Rootpath, Numab, Evolveimmune, Biontech, Immunocore, Glaxo Smith Kline, Adagene, Asher, Kanaph, iTEOS, Genocea, Trillium, Sapience, Targovax, Molecular Partners, Ontario Institute for Cancer Research, Jazz Pharmaceuticals, Gilead, Innate pharma, Tessa, Stcube, Oncosec, Regeneron, Astra Zeneca, Agenus, Idera, Apexigen, Verastem, Rubius, Genentech-Roche, Boston Pharmaceuticals, Servier, Dragonfly, Boehringer Ingelheim, Nektar, Pieris, Abbvie, Zelluna, and Seattle Genetics. Author MS has stock in: Johnson and Johnson, Glaxo-Smith Kline. Author MS has stock options in: Adaptive Biotechnologies, Amphivena, Intensity, Actym, Evolveimmune, Nextcure, Repertoire, Oncohost, Rootpath, Asher. Author SW has consulted for Array Biopharma and Magellan Rx. Institutional research funds have been granted by Bristol-Myers Squibb and Apexigen (HK, SW) and Merck (HK).

The remaining authors declare that the research was conducted in the absence of any commercial or financial relationships that could be construed as a potential conflict of interest.

## Publisher’s Note

All claims expressed in this article are solely those of the authors and do not necessarily represent those of their affiliated organizations, or those of the publisher, the editors and the reviewers. Any product that may be evaluated in this article, or claim that may be made by its manufacturer, is not guaranteed or endorsed by the publisher.

## Supplementary Material

Supplementary Figure 1 Cumulative incidence of ICI-induced hypophysitis in patients with melanoma over time in months.

Supplementary Figure 2 Serial free testosterone values drawn from male patients on immune checkpoint inhibitors pre-hypophysitis (baseline), at the time hypophysitis was diagnosed, and post-hypophysitis (POST #1 and POST #2).
