# Supplementary figures and images for: Immune Checkpoint Inhibitor-Induced Hypophysitis and Patterns of Loss of Pituitary Function
Source: Front Oncol. 2022 Mar 8;12:836859. doi: 10.3389/fonc.2022.836859 (PMC8958012; doi:10.3389/fonc.2022.836859)

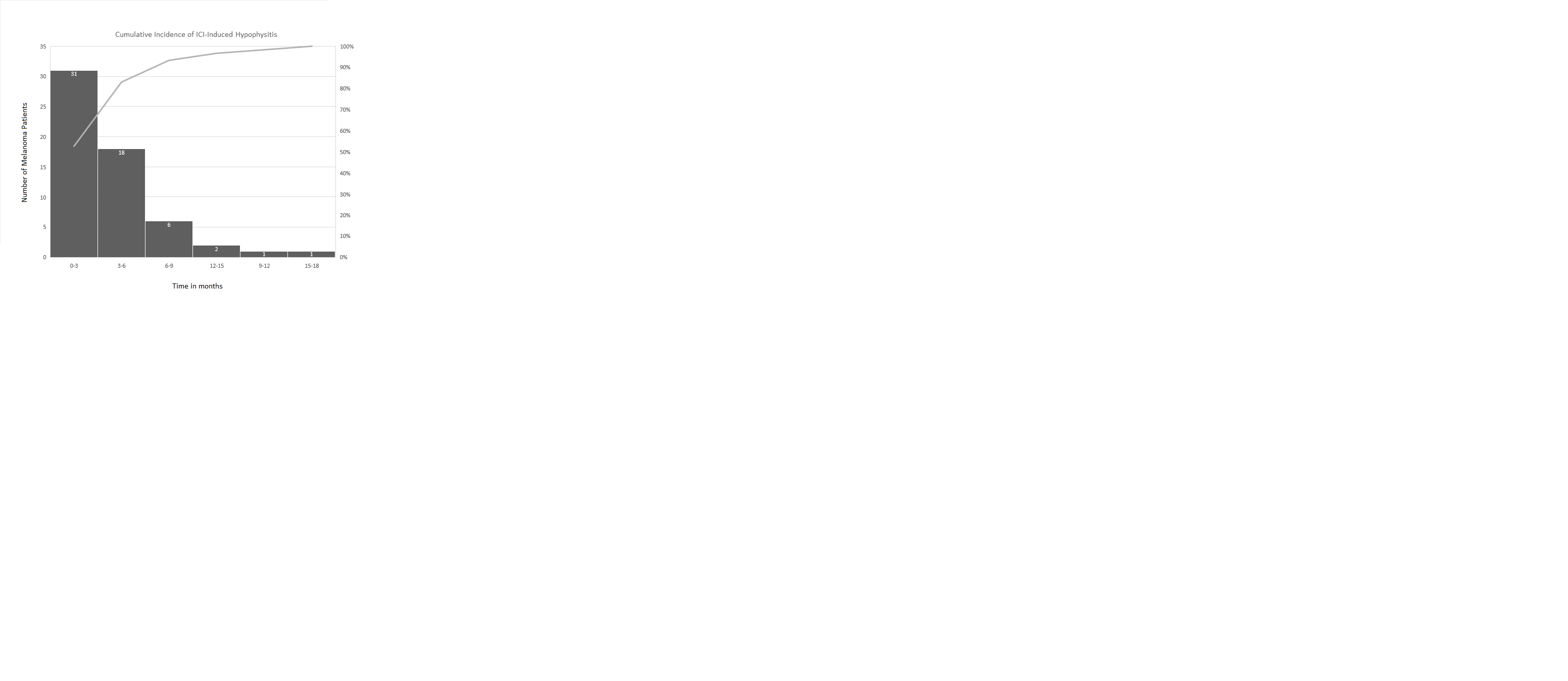

Supplement: Supplementary Figure 1 — Cumulative incidence of ICI-induced hypophysitis in patients with melanoma over time in months. [file Image_1.tif]

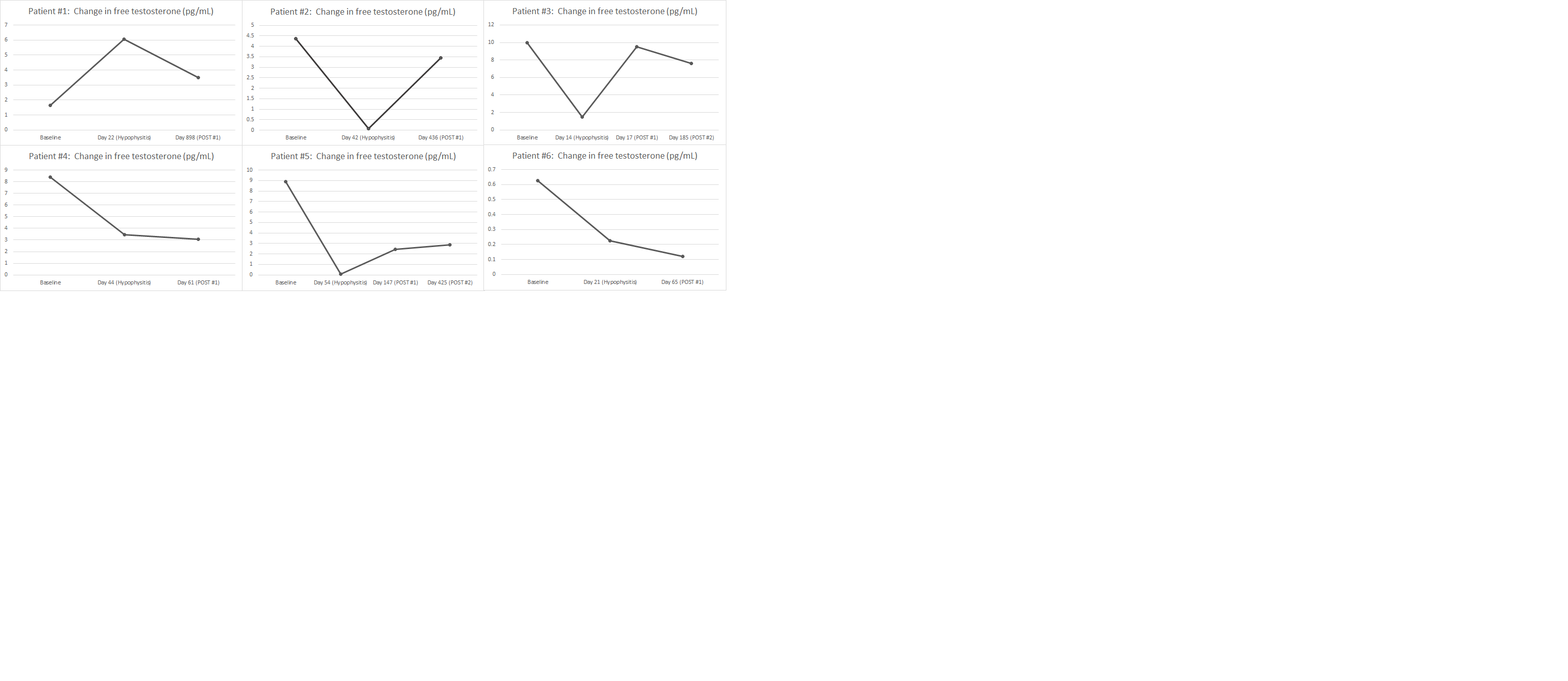

Supplement: Supplementary Figure 2 — Serial free testosterone values drawn from male patients on immune checkpoint inhibitors pre-hypophysitis (baseline), at the time hypophysitis was diagnosed, and post-hypophysitis (POST #1 and POST #2). [file Image_2.tif]
