# Supplementary material for: Immune Checkpoint Inhibitor-Induced Hypophysitis and Patterns of Loss of Pituitary Function
Source: Front Oncol. 2022 Mar 8;12:836859. doi: 10.3389/fonc.2022.836859 (PMC8958012; doi:10.3389/fonc.2022.836859)
Supplement: Supplementary file 4 [file Table_2.docx]

**Supplementary Data and Methods:**

Fifty-seven patients with melanoma had tumor profiling performed with the following mutations reported:

| **Common Melanoma Mutations (n=57)** | **n** | **%** |
| --- | --- | --- |
| Tumor profiling not performed | 1 | - |
| BRAF mutant | 23 | 40% |
| NRAS mutant | 6 | 11% |
| BRAF WT and NRAS WT | 28 | 47% |
| GNAQ/GNAS mutant | 4 | 7% |

Tumor profiling was performed using various methods, often either TaqMan or next-generation sequencing.
